# HIV-1 mutational pathways under multidrug therapy

**DOI:** 10.1186/1742-6405-8-26

**Published:** 2011-07-27

**Authors:** Glenn Lawyer, André Altmann, Alexander Thielen, Maurizio Zazzi, Anders Sönnerborg, Thomas Lengauer

**Affiliations:** 1 Department of Computational Biology, Max Planck Institute for Informatics, Saarbrücken, Germany; 2 Department of Statistical Genetics, Max Planck Institute of Psychiatry, Munich, Germany; 3 Department of Biotechnology, University of Siena, Siena, Italy; 4 Division of Infectious Diseases, Karolinska Institute, Stockholm, Sweden

## Abstract

**Background:**

Genotype-derived drug resistance profiles are a valuable asset in HIV-1 therapy decisions. Therapy decisions could be further improved, both in terms of predicting length of current therapy success and in preserving followup therapy options, through better knowledge of mutational pathways- here defined as specific locations on the viral genome which, when mutant, alter the risk that additional specific mutations arise. We limit the search to locations in the reverse transcriptase region of the HIV-1 genome which host resistance mutations to nucleoside (NRTI) and non-nucleoside (NNRTI) reverse transcriptase inhibitors (as listed in the 2008 International AIDS Society report), or which were mutant at therapy start in 5% or more of the therapies studied.

**Methods:**

A Cox proportional hazards model was fit to each location with the hazard of a mutation at that location during therapy proportional to the presence/absence of mutations at the remaining locations at therapy start. A pathway from preexisting to occurring mutation was indicated if the covariate was both selected as important via smoothly clipped absolute deviation (a form of regularized regression) and had a small p-value. The Cox model also allowed controlling for non-genetic parameters and potential nuisance factors such as viral resistance and number of previous therapies. Results were based on 1981 therapies given to 1495 distinct patients drawn from the EuResist database.

**Results:**

The strongest influence on the hazard of developing NRTI resistance was having more than four previous therapies, not any one existing resistance mutation. Known NRTI resistance pathways were shown, and previously speculated inhibition between the thymidine analog pathways was evidenced. Evidence was found for a number of specific pathways between NRTI and NNRTI resistance sites. A number of common mutations were shown to increase the hazard of developing both NRTI and NNRTI resistance. Viral resistance to the therapy compounds did not materially effect the hazard of mutation in our model.

**Conclusions:**

The accuracy of therapy outcome prediction tools may be increased by including the number of previous treatments, and by considering locations in the HIV genome which increase the hazard of developing resistance mutations.

## Background

Antiretroviral treatment has turned infection with the Human Immunodeficiency Virus (HIV-1) into a manageable disease. Yet eventually the HIV variants circulating in the patient develop resistance to the applied drugs. In many cases, it is known which mutations give resistance to which drugs, allowing accurate prediction of therapy efficacy based on HIV genotyping [1], with generally good results [2,3]. Better understanding of which pre-existing mutations effect the development of resistance would further improve treatment, informing both choice of compounds for the current therapy and long-term strategies to maintain treatment options when the current therapy fails.

Reverse transcriptase inhibitors (RTIs) are the longest used and arguably the most important class of antiretrovirals. These compounds inhibit the reverse transcription of single-stranded viral RNA into double-stranded viral DNA suitable for incorporation into the host DNA. They are classified as either nucleoside (NRTIs), which incorporate into and terminate transcription of the viral DNA, or non-nucleoside (NNRTIs), which change the conformation of the RT polymerase into a non-functional state. RTIs are expected to remain a critical therapy component even as new classes of drugs, such as entry and integrase inhibitors, are added to the anti-HIV arsenal [4].

Accordingly, a great deal of work has investigated development of RTI resistance. Many RTI resistance mutations are known to occur in clusters [5]. Two of the most studied NRTI clusters are the thymidine analog resistance mutations, TAM-1 (41L, 210W, 215Y) and TAM-2 (67N, 70R, 215F, 219E/Q), [6]. which show evidence of appearing in ordered sequence [6,7]. Less evidence supports pathways to NNRTI resistance, which can arise from a single mutation [8] with little impact on viral fitness [9-11]. Data from clinical trials of efavirenz (an NNRTI), however, suggested that mutation at location 103 preceded mutation at locations 100, 101, 108, and 225 [12,13].

Standard of care generally dictates two NRTIs supplemented with additional compounds which may include an NNRTI. Understanding of the development of resistance under such multidrug regimes is far from complete [14,15]. It has been shown that subjects with NNRTI resistance were at greater hazard of developing NRTI resistance, and vice versa [16], but not which specific factors explained this. Several sources have indicated interactions and other forms of crossplay between NRTI and NNRTI resistance mutations, but have not demonstrated clear pathways [4,17].

Many of the mutations which commonly occur during therapy do not have a known, direct connection with drug resistance. In the data studied here, 45 different locations frequently harbored mutations at therapy start; 32 of these are not on the International AIDS Society list of RTI resistance mutations [18]. Patterns within these other mutations may underly the just commented on interplay, lending high interest to pathways leading from commonly mutant locations to known resistance sites.

We place the question of identifying mutational pathways in a survival analysis framework. A mutational pathway from (genetic) location *a *to *b *is signalled if mutation at location *a *alters the hazard of mutation at location *b*. Survival analysis extends the specificity of investigations based on co-occurrence of mutations (i.e. [4,7,17]) by indicating both excitatory and inhibitory influences, incorporating temporal dynamics, and making full use of the data despite the abundant censoring. The framework further allows for control of nuisance parameters which are inherent in clinical data. In the current case, the most important of these is having a high number of previous therapies. While other techniques from survival analysis have been previously applied to RTI resistance [11,13,16], and protease inhibitor resistance [19], this is, to our knowledge, the first use of such methods to directly addresses the question of specific mutational pathways.

We tested for pathways between known resistance sites, and also for pathways between commonly mutant locations. Pathways were signalled by a two stage filtering process. For a given mutational site, we first applied smoothly clipped absolute deviation (SCAD) [20], a form of regularized regression, to identify a subset of pre-existing mutations which showed evidence of influencing the hazard of mutation at that site. These were further screened via standard significance testing to identify those with strong evidence for effect. The model also tested for effects associated with the clinical variables. Longitudinal data were available from the EuResist database [21]. EuResist maintains, to our knowledge, the largest HIV resistance database available for public research.

## Methods

### Subject material

Subject material for this study was drawn from the EuResist database [21]. The EuResist project integrates viral genotypes, therapy, and patient data collected by hospitals throughout Europe, notably from Italy, Germany, Sweden, Belgium, Spain, Portugal, and Luxembourg. Our study was based on therapy records which contain genotypes recorded both at therapy start and before therapy end, and which included an RTI. While the EuResist database was not designed with this desiderata, the 2010-01-26 release contains 1981 RTI-based therapies for which HIV genotype was recorded up to three months prior to therapy start, and a second genotype recorded before the end of said therapy. These therapies represent 1495 unique subjects. Two hundred and seven subjects appear twice, and 102 subjects appear multiple times. Table 1 lists the ten most frequently prescribed combinations of RTIs; the full list is given in the supplement [see Additional file 1, Table S1].

**Table 1:** Therapy profiles

Therapy profiles			
**Compounds**	**N**	**Duration**	**# Previous**

3TC AZT	259	549 (21,3515)	3 (0,28)

d4T DDI	149	553 (40,3291)	5.2 (0,19)

TDF FTC	123	284 (28,1122)	5.9 (0,25)

3TC d4T	115	646 (19,3508)	4.5 (0,18)

3TC TDF	96	311 (27,1360)	7.4 (0,20)

3TC DDI	61	590 (43,2268)	8.6 (1,37)

3TC ABC	52	397 (40,1140)	6.5 (0,18)

AZT	50	379 (1,1939)	2 (0,12)

3TC ABC AZT	50	437 (28,1423)	4.6 (0,19)

AZT DDI	49	413 (57,1408)	5.1 (0,17)

The data in the current study contains 119 unique combinations of reverse transcriptease inhibitors. The 10 most common combinations are listed here, along with the number of subjects receiving them, mean (range) duration in days, and mean (range) number of previous therapies when administered. Note that these therapies may also have included protease inhibitors. The full table is given in the supplement.

The outcome measure of the current study was the presence of mutation at a second genotype taken before therapy end. The distribution of the time delay between the first and second genotyping was approximately equally across the different risk groups [see Additional File 1, Figure S1]. The use of a second genotyping is subtly, but crucially, distinct from using time of therapy failure as an outcome measure. Twenty percent of the therapies were ongoing at the time the second genotype was recorded. Further, the EuResist database defines a therapy based on the compounds given. Therapies are considered to end when any compound in the therapy is added or removed, regardless of virological suppression. Often the cause is therapy change is not recorded. Table 2, therapy stop causes, indicates that 51% of the current therapies do not have a recorded stop cause. Only 19% of the second genotypings are unequivocally associated with therapy failure.

**Table 2:** Therapy stop causes

Therapy stop causes		
**Cause**	**count**	**percent**

Unknown	1026	0.52

Ongoing	398	0.20

Failure	372	0.19

Side effects	67	0.03

Change of therapy	57	0.03

Adherence	38	0.02

Supervised Interruption	23	0.01

TOTAL	1981	

EuResist defines a therapy based strictly on the compounds included. Any change in compounds is recored as a therapy change. The reason for therapy change was recorded for 75% of the records included here, and are listed in the database under the following categories.

All genotypes are population sequences reflecting the consensus HIV-1 genotype at the time of measurement. Subject demographics (shown in Table 3) are heterogeneous, and all major risk groups are well represented. The median number of previous therapies is 4, and ranges from no previous treatments (249 subjects) to 37 previous treatments (1 subject). The reason for including therapy naive subjects is that a pathway is defined by an increase in risk of developing a resistance mutation based on pre-existing mutations. Including therapy naive subjects in the model gives a better estimate of the baseline hazard estimate.

**Table 3:** Patient profile

Patient demographics		
Age 1st genotyping	39.7 years	± 9.3

Gender (M/F)	1054 M	433 F

Num prev therps	4 (median)	0-32 (range)

Days between genotypings	485 (mean)	639 (variance)

**Risk group:**		

Heterosexual	450	30%

Homo/bisexual	367	25%

IVDA	372	25%

Vertical transmission	33	2%

Blood products	25	2%

Other/unknown	245	16%

Background data on the subjects included in the study. When a subject has contributed multiple therapy records to the study, the demographic information is taken from the first therapy included.

### Binarization and locations (codons) considered

**Figure 1:**
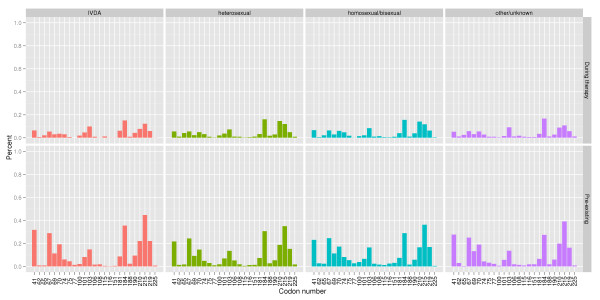
**Frequency of resistance mutations**. The percentage of therapies which developed mutation during therapy at the indicated location (top), and which had a preexisting mutation at the indicated location (bottom), by risk group. The patterns are consistent across the different risk groups. Locations are codons associated with significant resistance to one or more RTI compounds, as reported in the 2008 International AIDS Society listing [18].

**Figure 2:**
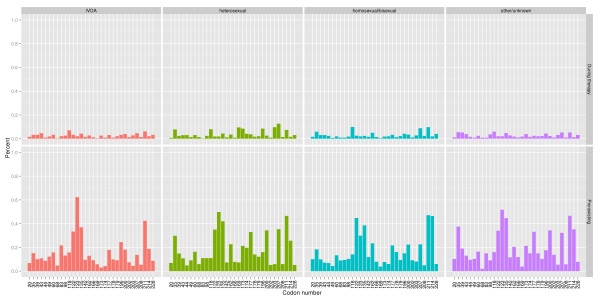
**Frequency of common mutations**. The percentage of therapies which developed mutation during therapy at the indicated location (top), and which had a preexisting mutation at the indicated location (bottom), by risk group. The patterns are consistent across the different risk groups. Locations are codons which were mutant at the start of 5% or more of the therapies, but which are not included on the International AIDS Society list of resistance mutations [18].

This study investigated locations (or codons) known to harbor RTI resistance mutations and locations which were commonly mutant in the EuResist data. Known resistance sites were drawn from 2008 International AIDS Society list of mutations associated with antiretroviral drug resistance [18]. This included the following locations: NRTI: 41, 62, 65, 67, 69, 70, 74, 75, 77, 115, 116, 151, 184, 210, 215, 219 and NNRTI: 100, 101, 103, 106, 108, 181, 188, 190, 225. Commonly mutant locations were defined as those which were mutant at the start of 5% or more of the therapies studied here, namely: 20, 35, 39, 41, 43, 44, 49, 60, 67, 68, 69, 70, 74, 83, 98, 101, 103, 118, 122, 123, 135, 142, 162, 166, 169, 173, 174, 177, 178, 179, 181, 184, 190, 196, 200, 202, 203, 207, 208, 210, 211, 214, 215, 219, and 228. Figure 1 (known resistance) and Figure 2 (commonly mutant) show the frequency of mutation at these locations, both at therapy start and at the second genotyping, for each of the major patient risk groups.

During this investigation only the location was specified and not the amino acid substitution. The assumption is that mutations detectable by population sequencing would be heavily influenced by treatment history. Binarization offered several additional advantages. It simplifies ambiguities arising from the genotyping method. Further, the study included commonly mutant locations not necessarily listed as important to resistance and thus with little literature support to decide which substitutions were relevant. Binarization allowed these locations to be treated identically to the known resistance mutations.

A location was considered to have a preexisting mutation if its genotype at therapy start did not completely agree with the wild type. For example, a location with wild-type "M" which showed the mixture "MV" at therapy start would be coded as a mutation. A mutation was considered to have occurred during therapy if the observed sequence did not contain an amino acid observed in the preexisting sequence and was not wild type. A preexisting "ML" which changed to "L" during the course of therapy would not be considered a mutation, while a change to "R" would be.

### Identifying mutational pathways-basic framework

Given a location of interest *c*, the goal was to identify other locations where preexisting mutations significantly altered the hazard of developing a mutation at *c*. The influence could be either inhibitory or excitatory. The question was formulated in terms of the Cox proportional hazards model using regressors to signal the presence/absence of preexisting mutations and to control for nuisance parameters. Formally,(1)

where *hc_i_*(*t*) is the hazard that location *c *becomes mutant in subject *i *during therapy and *hc*_0_(*t*) is the baseline hazard of mutation at *c*. The Cox model does not require specification of *hc*_0_(*t*), which is integrated out during the model fitting. A preexisting mutation at location *j^th ^*in subject *i *is coded by *p*_1_,*_i _*... *p*_*j*,*i*_. Having the median or fewer previous therapies was coded by *nuis*_*a*,*i*_. Other potential nuisance factors, such as the total number of mutations, viral resistance to the therapy compounds, and treatment start year were deemed non-informative in preliminary investigations. An event was signalled when location *c *was mutant in subject *i *at the second genotype but not the first. The time to event was the number of days between the two genotypes. While the direction of effect is likely to be correct, this simplifying assumption regarding time to event implies that estimates of magnitude should be regarded with caution.

Within this framework, identification of pathways reduces to a variable selection problem; selecting those regressors with strong evidence of effect on the hazard of developing the target mutation. We first filtered the list of potential regressors using smoothly clipped absolute deviation (SCAD) [20]. SCAD is a form of regularized regression, similar to the LASSO, but with the added benefit that the regularization parameter scales with the magnitude of the regression coefficient. The regressors included in the best SCAD model were then tested for significance using the Wald estimate, and those with *p <*0.01 were deemed to have sufficient evidence to suggest a pathway.

While each individual model could only detect one-step pathways (i.e. pre-existing mutation at locations *p*_3 _and *p*_7 _increased the hazard of mutation at *c*), fitting the model to each of the candidate regressors in turn produces an adjacency matrix which can be viewed as a directed graph allowing multi-step pathways. We first searched for pathways among known resistance sites by considering the combined list of NRTI and NNRTI resistance locations. We then searched for pathways in our list of commonly mutant locations.

Statistics and figures were created in the R software environment, version 2.7.1 [22]. SCAD was implemented using the R package SIS [23]. Cox model fitting and the Wald estimate were performed using the R package survival [24]. Visualization was aided by the packages ggplot2 [25] and igraph [26].

### Identifying mutational pathways - specific models

The basic model identifies pre-existing mutation with strong evidence for effect on the hazard of developing mutation at one specific target location *c*. Given this location, therapy data is restricted to those at risk of developing mutation at *c*, that is, those whose HIV genotype did not exhibit mutation at *c *at the start of treatment. When *c *was an NNRTI resistance location, therapies were further restricted to those receiving an NNRTI. Note that all of the therapies under consideration included NRTIs. Candidate regressors were also dependent on *c*. Obviously *c *itself could not be a candidate. Further, some locations never exhibited a preexisting mutation in at-risk therapies, and were dropped to prevent convergence issues. Finally, if data is not available on a specific pre-existing mutation for more than 10 of the at-risk therapies, it was dropped from the model.

## Results and Discussion

The single factor which most consistently influences the hazard of mutation at locations with known involvement in NRTI resistance is the number of previous therapies. The median number of previous therapies in the data studied here is 4. Therapies for patients with 4 or less previous therapies are associated with less than half the risk of developing RT resistance. The effect is observed at 9 out of 16 locations associated with NRTI resistance: codons 65, 67, 69, 70, 74, 115, 210, 215, and 219. The median hazard ratio is 0.37 (ranging from 0.15 to 0.52), with the lowest 95% confidence bound at 0.06 and the highest at 0.81. This effect is not due to the presence of more known resistance mutations in patients with a large number of previous therapies, as known resistance mutations were regressed out by the model. In addition, further testing indicated that genotypically estimated viral resistance has negligible effect in our models. The finding could, however, represent the accumulation of mutations in regions about which little is known because they are rarely sequenced. For example, the first investigation of mutations in the connection and ribonuclease H domains of RT has shown that such mutations strongly influence AZT resistance in combination with the TAM pathways [27].

The study clearly substantiates the well established TAM pathways. The TAM-1 pathway is demonstrated by the observation that mutation at location 215 increases the hazard of mutation at location 41. The TAM-2 pathway is supported by finding that mutation at location 67 increases the hazard of mutation at locations 70 and 219, and that mutation at 70 increases the hazard at 219. All of these pathways involve an estimated three-fold increase in hazard. (Estimated hazard ratios for all indicated mutational pathways which end at known resistance sites are given in Tables 4 and 5. Hazard ratios for pathways which end at commonly mutant locations are listed in the supplement [see Additional file 1, Table S2]. Our results concur with biological assays suggesting that 215 precedes 41 in development of the TAM-1 pathway, [6], and that 67N and 70R are the first mutations to appear in the TAM-2 pathway [27]. The pathway order as determined by the current model largely agrees with that determined by the mutagenetic tree model both as applied to the current data and as in the original publication [7].

**Table 4:** Hazard Ratios for pathways between known resistance locations

Hazard ratios between known resistance locations		
**Pathway**	**Hazard**	**Confidence Bounds**

41 → 108	7.54	(2.2, 25.8)

67 → 70	3.52	(2.08, 5.98)

67 → 190	3.19	(1.41, 7.21)

67 → 219	2.97	(1.72, 5.14)

70 → 210*	0.33	(0.15, 0.75)

70 → 219	2.68	(1.6, 4.47)

74 → 100	12.92	(3.48, 47.95)

77 → 103	7.76	(2.6, 23.16)

115 → 106	17.51	(1.26, 243.88)

116 → 62	27.81	(8.73, 88.58)

151 → 116	237.67	(34.71, 1627.63)

181 → 65	3.28	(1.22, 8.81)

184 → 181^†^	1.77	(1.07, 2.92)

184 → 210	0.27	(0.17, 0.42)

190 → 184	1.84	(1.15, 2.95)

210 → 70	0.09	(0.03, 0.23)

215 → 41	3.23	(2.15, 4.85)

215 → 65	0.06	(0.01, 0.28)

Estimated hazard ratios and 95% confidence bounds for all pathways between known resistance locations with *p *< 0.01. Two additional pathways, which were remarked on in the text, are also included. Estimation was done using the survival [24] package of the R software environment [22].

*p = 0.011 ^†^*p*= 0.019

**Table 5:** Hazard Ratios for pathways from commonly mutant locations to known resistance locations

Hazard ratios; common to resistance locations		
**Pathway**	**Hazard**	**Confidence Bounds**

43 → 103	1.92	(0.85, 4.35)

49 → 103	0.46	(0.20, 1.07)

67 → 70	4.66	(2.17, 9.99)

67 → 190	3.31	(1.70, 6.42)

68 → 184	1.93	(1.21, 3.08)

70 → 103	1.01	(0.49, 2.08)

70 → 181	0.37	(0.15, 0.92)

70 → 219	2.76	(1.61, 4.74)

74 → 184	1.84	(1.07, 3.17)

118 → 219	1.62	(0.83, 3.14)

135 → 210	0.38	(0.25, 0.59)

142 → 67	1.89	(1.13, 3.16)

162 → 70	1.89	(1.08, 3.31)

179 → 103	1.98	(0.97, 4.02)

184 → 181	1.92	(1.13, 3.27)

184 → 190	0.73	(0.39, 1.39)

196 → 103	1.87	(1.14, 3.05)

196 → 190	1.90	(0.92, 3.92)

196 → 210	0.47	(0.24, 0.93)

200 → 190	1.76	(0.92, 3.35)

210 → 70	0.12	(0.04, 0.34)

211 → 210	0.46	(0.31, 0.68)

214 → 69	2.69	(1.44, 5.03)

215 → 41	3.12	(2.05, 4.75)

Estimated hazard ratios and 95% confidence bounds for pathways leading from commonly mutant locations to locations with known association to RTI resistance. Slight discrepancies in estimated hazard ratios between this table and Table 4 are due to a different choice of regressors used to fit the model. Estimation was done using the survival [24] package of the R software environment [22].

Inhibition of TAM-2 by TAM-1 is also observed. Mutation at location 210 is associated with a tenfold reduction in the risk of mutation at 70. A similar effect is witnessed in the reverse direction, though the reduction is only threefold, and the significance level is just above threshold (*p *= 0.011). The to date most thorough report on the TAM pathways [6] presented speculative evidence that TAM-1 inhibits TAM-2. Independent support for this inhibition was presented by Sing et. al [28]. Other studies, however, have reported that some patients develop mutations in both TAM clusters, or switching from TAM-1 to TAM-2 [29].

No pathways are seen between NNRTI resistance locations. This was not unexpected, as in vitro investigations suggest that resistance to most NNRTIs can result from a single mutation [8]. This evidence is corroborated by a Bayesian analysis of combinatorial mutation patterns which indicates that interactions among mutations granting nevirapine (an NNRTI) resistance were very weak [30]. Pathways have been suggested, however, in data from a clinical trial of efavirenz [12,13].

The method also indicated several cross-class resistance pathways. Specific pathways from NRTI to NNRTI resistance included the following, all of which showed multi-fold increase in hazard: 41 → 108, 67 → 190, 74 → 100, and 77 → 103. Previous work has observed that mutation at 74 is associated with increased frequency of NNRTI failure [4]; mutation at location 100 grants resistance to most NNRTIs [18]. Some evidence also suggests that the L74V mutation compensates loss of viral fitness incurred by the double NNRTI resistance mutations L100I + K103N [31].

**Figure 3:**
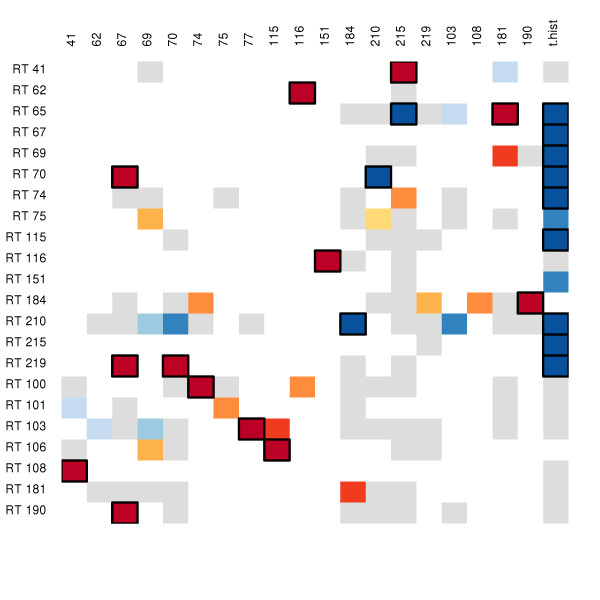
**Adjacency matrix of pathways within RTI resistance locations**. Columns are pre-existing mutations, rows are the outcome mutation. The matrix has been reduced to only include rows or columns with at least one detected effect. Colored cells are those which were suggested by SCAD. Grey cells had *p *> 0.05; the remaining cells are colored based on p-value, with darker colors indicating lower p-values. Red colors indicate a pre-existing mutation was associated with an increase in hazard, blue indicates a reduction. A box has been drawn around cells with *p *< 0.01.

**Figure 4:**
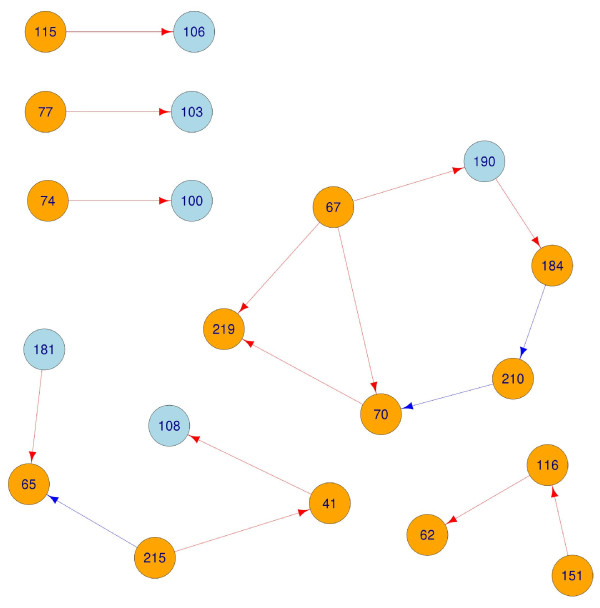
**RTI resistance pathways**. The adjacency matrix from Figure 1 (thresholded at *p *< 0.01) rendered as a graph. Red pathways indicate an increase in hazard, blue a reduction. Orange nodes are locations hosting NRTI resistance mutations, blue nodes are NNRTI locations.

A pathway was suggested from 184 to 181, though the associated p-value (0.019) is above our threshold. This finding is disconcerting, as mutation at 184 is one of the most common routes to NRTI resistance, and mutation at 181 grants resistance to all NNRTIs [18]. Specific NNRTI to NRTI resistance pathways are 181 → 65 and 190 → 184. Bayesian networks have suggested robust dependencies between NRTI mutations at 65, 74, 75, and 184 and NNRTI mutations at 100, 181, 190, and 230 [17], though the pathways suggested by the current work are not explicitly implicated in [17]. The adjacency matrix describing the pathways indicated in the current study is given in Figure 3, a network representation is given in Figure 4.

Survival analysis has shown that having any (N)NRTI resistance mutation increases the hazard of developing a mutation in the other class, [16]. We note that Healy et al.'s findings of general dependence showed stronger effect sizes than ours. This slight divergence could be dependent on the selection of subjects. Most of Healy et al.'s subjects had few previous treatments, while the EuResist subjects had failed a median of four previous therapies. As the EuResist subjects had a number of accumulated mutations, the risk profile of mutations which could arise in these subjects is likely to differ substantially from Healy et al.'s subjects. The evolutionary dynamics might also differ between the two groups. The accumulated mutations in the HIV variants circulating in patients with a long treatment history are likely to have reduced the virus's replicative capacity [32]. Differences could also be specific to viral subtype. We note that neither Healy et. al. nor the report of the clinical trial which supplied their data provide subtype information. Finally, Healy et al.'s data came from a prospective study, whereas the EuResist data is retrospective.

**Figure 5:**
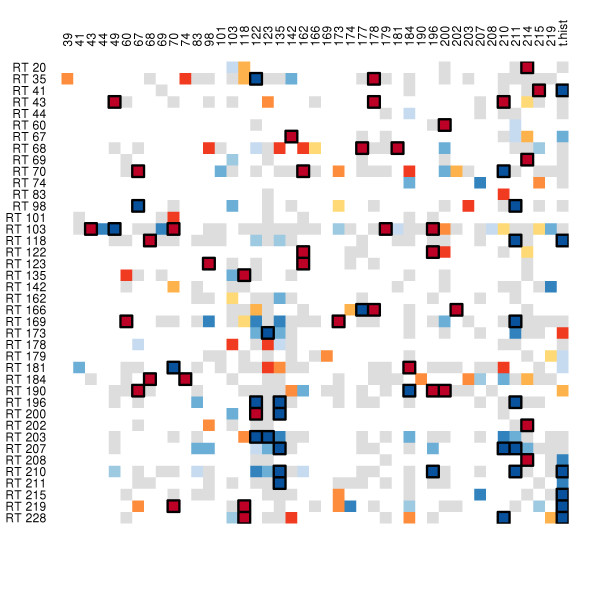
**Adjacency matrix of pathways within commonly mutant locations**. Columns are pre-existing mutations, rows are the outcome mutation. The matrix has been reduced to only include rows or columns with at least one detected effect. Colored cells are those which were suggested by SCAD. Grey cells had *p *> 0.05; the remaining cells are colored based on p-value, with darker colors indicating lower p-values. Red colors indicate a pre-existing mutation was associated with an increase in hazard, blue indicates a reduction. A box has been drawn around cells with *p *< 0.01.

**Figure 6:**
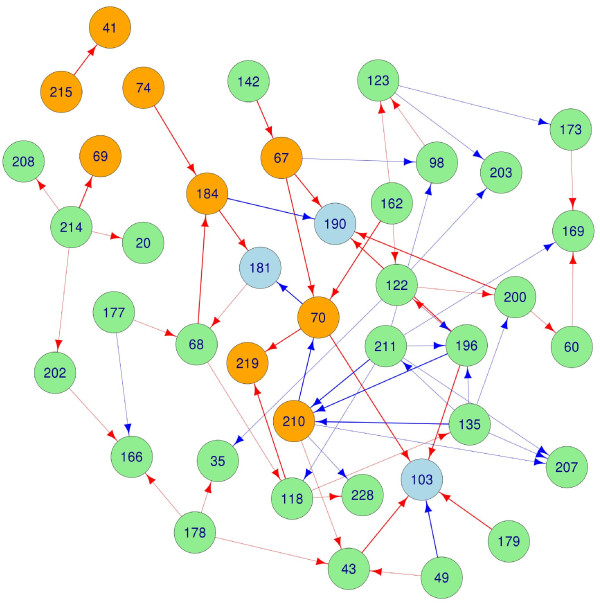
**Pathways in commonly mutation locations**. The adjacency matrix from Figure 3 (thresholded at *p *< 0.01) rendered as a graph. Red pathways indicate an increase in hazard, blue a reduction. Edges leading into or out from known resistance sites have been made thicker. Orange nodes are locations hosting NRTI resistance mutations, blue nodes are NNRTI locations, green nodes are not on the International AIDS society list.

Commonly mutant locations were defined as those locations which hosted a mutation at the start of at least 5% of the therapies analyzed in this study. Of the 45 locations which met this criteria, 13 are known to harbor resistance mutations. Fifteen edges lead out from known resistance sites. Five of these connect to locations not associated with resistance. The patterns observed above in the known resistance mutations are mostly preserved in this list. This should not be regarded as an independent observation. Though the candidate regressors are different, the data is the same. The adjacency matrix describing pathways in commonly mutant locations is given in Figure 5, a network representation is given in Figure 6.

Pathways which lead to known resistance sites could prove informative in predicting the development of (further) resistance. A number of pathways to NRTI resistance sites begin from locations not listed as providing RTI resistance, though the estimated increase in hazard is in general lower than that observed between known resistance mutations (see Table 5). Their influence suggests that therapy outcome prediction engines could be improved by incorporation of the following pathways: 142 → 67, 214 → 69, and 162 → 70. It was also observed that 68 → 184, and that mutation at either 177 or 181 increases the hazard of 68. This final observation suggests an indirect 181 → 68 → 184 pathway from NNRTI to NRTI resistance. An inhibitory pathway was also identified, with mutation at 135 reducing the hazard of mutation at 210 by a factor of 0.38 (95% confidence bounds of 0.25, 0.59).

Several pre-existing mutations are associated with increased hazard for mutation granting NNRTI resistance, concurring with previous research suggesting that pathways to NNRTI resistance may start from previously unsuspected mutations [4]. Notably, mutation at any of 43, 179,or 196 increases the hazard of mutation at 103.

196 or 200 increase the hazard at 190. Mutation at location 103 or 190 grants strong resistance to both EFV and NVP. This again suggests that consideration might be given to mutations at locations 43, 179, 196, or 200 before prescribing these NNRTIs. Location 43 could play a part in NRTI to NNRTI resistance, since 210 → 43 → 103. The 184 → 181 pathway suggested in the known resistance sites was again observed, and now with sufficient evidence to pass our threshold.

Several mutations seemed to decrease the hazard of NNRTI resistance. Notably, mutation at location 70 (part of the TAM-1 complex granting NRTI resistance) strongly inhibits mutation at location 181. Location 184 was observed to slightly inhibit mutation at location 190.

This work is, as far as we know, the first to fully employ the Cox model to identify specific mutational pathways. The proportional hazards approach is more sophisticated than methods which do not include time to event or censored events and which cannot control for nuisance parameters. Modeling was simplified, however, by assuming right censored data whereas interval censoring better describes the process generating the data. This simplifying assumption was required as we had no good indication of what an appropriate interval would be. It was justified in that the principle benefit of interval censoring is a more accurate estimate of effect size, not effect significance. Effect size did not play a role in our estimation of pathways.

Some evidence suggests that viral subtype also effects hazard of mutation. Several studies have observed a relationship between resistance mutations and viral subtype, as reviewed in [33] and [34]. A difficulty with such studies, however, is that the data on non-B subtypes tends to come from resource-limited regions where treatment regimes do not always meet European standards of best clinical care. This situation is likely to change as the demographics of the disease change; the UK Health Protection Agency now reports that the majority of UK infections are non-B [35]. Also, molecular dynamics suggest that different patterns of DNA synthesis in subtype C variants compared to subtype B increase the hazard of developing the K65R mutation [36]. Since subtype prevalence varies greatly by patient risk category, it is possible that analysis of subjects in the heterosexual risk group might demonstrate different pathways, or the same pathways with different hazard ratios, than observed in the full data. On the other hand, binarization of the data may have reduced this effect. The V106M resistance mutation may be preferred over V106A in subtype C [37]; the current study gives both of these events the same encoding. Preliminary analysis found subtype to be non-informative to the current model as applied to the EuResist data.

Data for the study came from a large observational database. This can lead to several forms of bias in the data. While we believe these effects to be small, they should be noted. The first bias deals with model assumptions. Our data was censored at the time of the second genotyping. This event is not necessarily independent from our outcome measure, the hazard of mutation. Dependence between the two, however, is difficult to ascertain. The bias would be strongest when the genotyping was conducted due to virological failure of the therapy, a factor which would increase the hazard of additional mutation. On the other hand, virological failure could be caused by the development of resistance mutations, which are present in the model and whose effect is therefore already included in the calculations.

The remaining biases are due to the clinical practices which underly the data gathering. For some, and perhaps most, of the therapies, genotyping was used to select treatment. Neither drug choices nor drug combinations were random, nor were the associations between drugs and resistance mutations. The EuResist database does not record which, if any, patients were left on failing regimens. Continuing a therapy in the presence of detectable viral loads is linked to increased hazard of mutation [38]. Finally, it should always be remembered that correlation does not imply causality. The underlying reason for an observed dependency could be influenced by shared inheritance from a founder virus or shared evolutionary pressures.

The current work did not investigate pathways to protease inhibitor (PI) pathways resistance as our primary interest was on cross-class RT mutational pathways. Nonetheless, some key work on pathways to protease inhibitor (PI) resistance should be noted. Many of the groups researching RTI resistance have also worked on PI resistance, and the development of statistical approaches has come from both endeavors. PI resistance mutations form clusters [19]. Temporal dependencies in HIV genetic changes in response to PIs have been indicated via Bayesian model selection [39]. Bayesian networks have been used to uncover direct influences between protein residues and treatment in clinical settings, and were able to determine the specific role of many resistance mutations against nelfinavir [40]. Phylogenetic approaches are also informative in determining pathways [13]. A cross-class pathway with promising clinical implications is a finding that patients with PI resistance are less likely to develop resistance to Bevirimat (a maturation inhibitor) than those who are PI-naive [41].

## Conclusions

This investigation found specific pathways both within and across drug classes. It was further found that the most consistent factor speeding the development of NRTI resistance was not any known mutation, but rather having failed more than four previous therapies.

## Competing interests

The authors declare that they have no competing interests.

## Authors' contributions

GL designed the study, performed the statistical analysis, and wrote the text. AA assisted with database issues, interpretation of results, and insight into HIV-1 drug resistance and drug resistance testing. AT assisted with literature suggestions, modifications to the methods, and interpret the results. MZ and AS participated in gathering the data and critically revised the manuscript. TL provided scientific guidance, critically read the text, and suggested points of interpretation. All authors read and approved the final manuscript.

## Supplementary Material

Additional file 1 **Supplementary materials**. This file contains Figure S1, showing the relationship between event times and risk group, Table S1, showing all therapy profiles in the current data, and Table S2, the estimated hazard ratios for all identified pathways between commonly mutant locations. Click here for file
