# Supplementary material for: HIV-1 mutational pathways under multidrug therapy
Source: AIDS Res Ther. 2011 Jul 27;8:26. doi: 10.1186/1742-6405-8-26 (PMC3162516; doi:10.1186/1742-6405-8-26)
Supplement: Additional file 1 — Supplementary materials. This file contains Figure S1, showing the relationship between event times and risk group, Table S1, showing all therapy profiles in the current data, and Table S2, the estimated hazard ratios for all identified pathways between commonly mutant locations. [file 1742-6405-8-26-S1.PDF]

HIV mutational pathways under multidrug therapy – Supplementary materials

GLENN LAWYER*
Department of Computational Biology
Max Planck Institute for Informatics, Saarbrücken, Germany

ANDRÉ ALTMANN
Department of Statistical Genetics
Max Planck Institute of Psychiatry Munich, Germany

ALEXANDER THIELEN
Department of Computational Biology
Max Planck Institute for Informatics, Saarbrücken, Germany

THOMAS LENGAUER
Department of Computational Biology
Max Planck Institute for Informatics, Saarbrücken, Germany

This supplement presents tables and figures which were not essential to the main text, but which offer a more detailed understanding of the findings. These include:

- Figure S1: Event times by risk ID
- Table S1: Therapy profiles
- Table S2: Hazard ratios for identified pathways between commonly mutant locations

Running title: HIV mutational pathways supplement

*To whom correspondence should be addressed.
Campus E1 4, 66123, Saarbrücken, Germany
Tel.: +49 681 9325 307 Fax: +49 681 9325 399
lawyer@mpi-inf.mpg.de

Figure S1 - Event times by risk ID

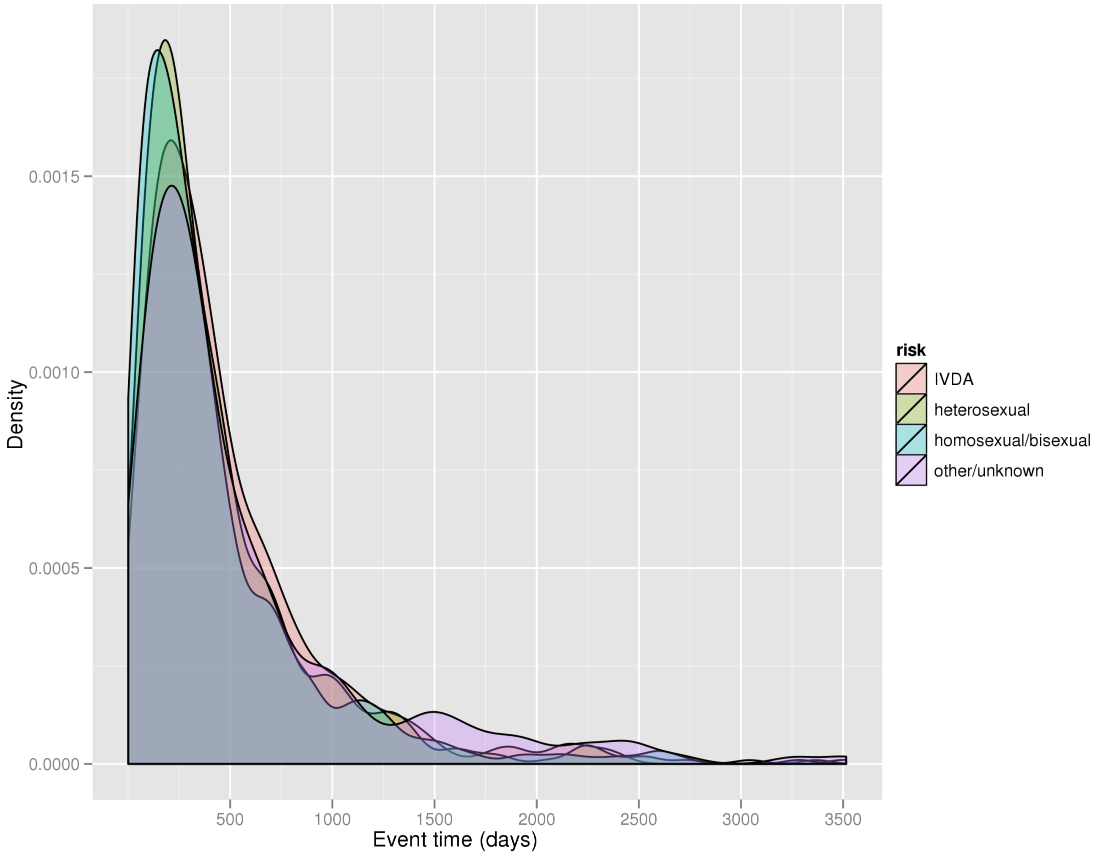

The distribution of the time between genotyping events had approximately the same distribution across the major risk groups.

Table S1 - Therapy profiles

The data in the current study represents 119 unique combinations of reverse transcriptease inhibitors. These are listed here, along with the number of subjects recieving them, mean (range) duration in days, and mean (range) number of previous therapies when administered. Note that these therapies may also have included protease inhibitors.

Therapy profiles			
Compounds	N	duration	prev. therps
3TC AZT	259	549 (21,3515)	3 (0,28)
d4T ddI	149	553 (40,3291)	5.2 (0,19)
TDF FTC	123	284 (28,1122)	5.9 (0,25)
3TC d4T	115	646 (19,3508)	4.5 (0,18)
3TC TDF	96	311 (27,1360)	7.4 (0,20)
3TC ddI	61	590 (43,2268)	8.6 (1,37)
3TC ABC	52	397 (40,1140)	6.5 (0,18)
AZT	50	379 (1,1939)	2 (0,12)
3TC ABC AZT	50	437 (28,1423)	4.6 (0,19)
AZT ddI	49	413 (57,1408)	5.1 (0,17)
3TC AZT NVP	43	714 (29,3043)	2.5 (0,12)
d4T	37	442 (23,2160)	5.7 (0,21)
3TC	37	462 (40,2520)	6.7 (0,21)
d4T TDF	34	274 (36,915)	6.6 (0,17)
ddI TDF	31	413 (28,1771)	7.8 (0,31)
AZT DDC	31	445 (42,1650)	1.4 (0,4)
d4T ddI NVP	29	494 (133,1270)	3.6 (0,10)
d4T ddI EFV	29	543 (73,2172)	4.2 (0,17)
3TC AZT EFV	29	562 (28,2590)	1.5 (0,8)
ABC d4T	27	434 (30,1595)	7.5 (1,20)
d4T NVP	23	311 (91,618)	4.6 (1,13)
ABC ddI	23	651 (63,3284)	8.6 (1,18)
TDF FTC EFV	22	292 (29,1207)	1.4 (0,6)
AZT TDF	21	387 (57,885)	7.3 (1,18)
ABC TDF	21	576 (60,2461)	6.3 (0,13)
3TC AZT TDF	19	493 (73,2626)	8.8 (0,20)
3TC d4T NVP	18	696 (90,2156)	3.2 (0,10)
3TC ABC AZT TDF	18	322 (92,943)	6 (0,13)
3TC TDF EFV	16	404 (43,1422)	4.2 (0,20)
TDF	15	388 (62,1323)	9.9 (1,21)
3TC ABC EFV	15	357 (51,1241)	5.4 (0,13)
ABC AZT	14	549 (56,1468)	6.5 (2,20)
ddI	13	325 (49,947)	5.8 (0,15)
ABC	13	194 (15,521)	3.1 (0,14)
3TC TDF NVP	13	377 (2,2062)	6.5 (1,16)
3TC ddI TDF	13	506 (68,1644)	10.3 (2,35)
d4T EFV	12	543 (112,1274)	4.9 (1,8)
ABC d4T EFV	12	614 (83,1288)	5.8 (1,12)
3TC NVP	12	683 (169,1311)	8.8 (1,19)

Therapy profiles			
Compounds	N	duration	prev. therps
EFV	11	579 (42,1778)	6.8 (1,12)
ABC d4T ddI	11	559 (29,1591)	6.7 (2,19)
3TC ABC d4T	11	369 (5,1262)	8.8 (2,17)
TDF EFV	10	525 (63,1660)	8.8 (4,16)
ABC ddI EFV	10	319 (33,1656)	4 (0,16)
3TC ddI EFV	10	557 (78,1509)	4.5 (0,18)
3TC ABC TDF	10	356 (19,787)	6.6 (0,22)
ddI TDF EFV	9	458 (30,1090)	3 (0,5)
d4T TDF EFV	9	458 (133,1024)	6.2 (2,13)
AZT ddI EFV	9	675 (21,1790)	4.8 (1,15)
3TC d4T EFV	9	576 (223,1307)	4.8 (1,10)
3TC d4T TDF	9	406 (67,1057)	4.3 (1,13)
3TC ABC ddI	9	438 (175,1280)	8.3 (2,15)
NVP	8	617 (105,1372)	6.6 (2,9)
ddI TDF NVP	8	264 (163,399)	5.6 (1,12)
d4T TDF NVP	8	368 (125,744)	8 (2,21)
d4T DDC	8	534 (89,2302)	2.8 (1,5)
AZT ddI NVP	8	541 (92,2147)	2 (0,4)
3TC d4T ddI	8	636 (91,2577)	8.6 (2,18)
ddI NVP	7	602 (126,1964)	9 (3,16)
ABC ddI TDF	7	433 (118,1195)	12.6 (2,30)
ddI EFV	6	903 (126,2226)	5.7 (3,8)
AZT TDF FTC	6	404 (178,604)	7.3 (0,19)
3TC AZT ddI	6	743 (165,1722)	10.7 (3,24)
d4T ddI TDF	5	591 (237,1089)	6.2 (2,10)
ABC EFV	5	436 (91,1177)	6 (1,8)
ABC ddI NVP	5	165 (91,229)	5.6 (1,16)
3TC EFV	5	199 (36,399)	13 (1,28)
3TC ddI NVP	5	479 (119,762)	6.8 (2,12)
3TC ABC d4T EFV	5	413 (200,790)	9 (0,18)
TDF FTC NVP	4	143 (72,217)	5.5 (0,12)
d4T TDF FTC	4	228 (88,462)	13.2 (7,25)
AZT EFV	4	323 (114,628)	6 (1,9)
AZT TDF EFV	4	318 (116,472)	4.5 (0,16)
3TC ABC AZT EFV	4	434 (69,659)	8.2 (3,13)
FTC	3	516 (167,890)	12.7 (10,16)
TDF NVP	3	489 (133,992)	8.7 (6,13)
TDF FTC TMC125	3	199 (36,371)	15.7 (5,22)
AZT NVP	3	344 (244,511)	2.3 (0,6)
AZT TDF NVP	3	279 (168,349)	3 (1,7)
AZT ddI TDF	3	372 (114,672)	12 (4,17)
ABC TDF NVP	3	415 (231,702)	8.7 (4,13)
ABC TDF EFV	3	311 (100,653)	5 (1,10)
ABC AZT TDF	3	231 (118,371)	6 (1,11)
3TC ABC NVP	3	736 (361,967)	2.7 (0,5)

Therapy profiles			
Compounds	N	duration	prev. therps
TMC125	2	154 (54,253)	11.5 (2,21)
ddI FTC	2	528 (329,727)	4.5 (2,7)
ddI TDF FTC	2	336 (299,374)	3 (3,3)
d4T DDC NVP	2	280 (175,386)	3.5 (3,4)
ABC NVP	2	368 (100,637)	9 (9,9)
ABC d4T TDF	2	403 (212,594)	11.5 (9,14)
ABC d4T ddI EFV	2	179 (149,209)	6 (3,9)
ABC AZT NVP	2	216 (210,222)	6.5 (1,12)
ABC AZT ddI	2	1442 (658,2226)	1.5 (1,2)
3TC d4T TDF NVP	2	678 (418,938)	10.5 (7,14)
3TC AZT TDF EFV	2	290 (240,339)	1 (1,1)
3TC AZT ddI EFV	2	374 (233,514)	7.5 (5,10)
3TC AZT DDC	2	270 (56,485)	2.5 (2,3)
3TC AZT d4T	2	350 (280,421)	8.5 (4,13)
3TC ABC AZT ddI	2	389 (365,413)	13 (12,14)
FTC EFV	1	487 (487,487)	8 (8,8)
TDF TMC125	1	250 (250,250)	14 (14,14)
d4T TDF FTC EFV	1	150 (150,150)	3 (3,3)
d4T ddI TDF NVP	1	272 (272,272)	13 (13,13)
AZT TDF FTC NVP	1	3 (3,3)	2 (2,2)
ABC EFV NVP	1	232 (232,232)	9 (9,9)
ABC FTC	1	168 (168,168)	13 (13,13)
ABC FTC NVP	1	251 (251,251)	34 (34,34)
ABC DDC	1	343 (343,343)	14 (14,14)
ABC AZT TDF NVP	1	336 (336,336)	2 (2,2)
ABC AZT DDC	1	435 (435,435)	13 (13,13)
3TC ddI TMC125	1	817 (817,817)	8 (8,8)
3TC ddI TDF EFV	1	87 (87,87)	4 (4,4)
3TC DDC	1	157 (157,157)	6 (6,6)
3TC d4T ddI EFV	1	558 (558,558)	2 (2,2)
3TC ABC TMC125	1	349 (349,349)	15 (15,15)
3TC ABC TDF EFV	1	293 (293,293)	6 (6,6)
3TC ABC DDC	1	89 (89,89)	12 (12,12)
3TC ABC AZT TDF NVP	1	186 (186,186)	17 (17,17)
3TC ABC AZT TDF EFV	1	57 (57,57)	9 (9,9)

Table S2 - Hazard ratios for identified pathways between commonly mutant locations

Estimated hazard ratios and 95% confidence bounds for pathways from all commonly mutant locations to locations with no known association to RTI resistance. Estimation was done using the `survival` package of the R software environment.

Hazard ratios; common to common locations		
49 → 43	2.34	(1.22, 4.49)
60 → 169	3.76	(0.99, 14.27)
67 → 98	0.33	(0.11, 0.95)
68 → 118	2.23	(0.99, 4.99)
98 → 123	2.61	(0.96, 7.13)
118 → 135	3.55	(1.67, 7.55)
118 → 228	2.42	(1.08, 5.43)
122 → 35	0.50	(0.28, 0.92)
122 → 196	0.52	(0.29, 0.96)
122 → 200	2.32	(1.35, 3.99)
122 → 203	0.43	(0.22, 0.84)
123 → 173	0.16	(0.07, 0.37)
123 → 203	0.32	(0.17, 0.60)
135 → 196	0.35	(0.20, 0.64)
135 → 200	0.35	(0.18, 0.67)
135 → 207	0.26	(0.14, 0.48)
135 → 211	0.45	(0.27, 0.73)
162 → 122	2.47	(1.19, 5.13)
162 → 123	1.83	(0.71, 4.70)
173 → 169	3.72	(1.03, 13.43)
177 → 68	4.62	(1.51, 14.10)
177 → 166	0.29	(0.08, 1.10)
178 → 35	1.94	(0.98, 3.85)
178 → 43	1.80	(0.97, 3.34)
178 → 166	2.69	(0.96, 7.51)
181 → 68	5.62	(1.26, 24.98)
196 → 122	2.39	(1.21, 4.70)
200 → 60	1.32	(0.73, 2.40)
202 → 166	3.03	(0.84, 10.88)
210 → 43	2.38	(1.20, 4.70)
210 → 207	0.68	(0.39, 1.19)
210 → 228	0.18	(0.06, 0.50)
211 → 98	0.70	(0.36, 1.34)
211 → 118	0.58	(0.38, 0.87)
211 → 169	0.09	(0.02, 0.40)
211 → 196	0.46	(0.27, 0.80)
211 → 207	0.34	(0.19, 0.58)
214 → 20	3.72	(1.57, 8.78)
214 → 202	3.66	(1.44, 9.31)
214 → 208	1.67	(0.81, 3.45)
